# Supplementary material for: Intervening on Global Emergencies: The Value of Human Interactions for People’s Health
Source: Behav Sci (Basel). 2023 Sep 2;13(9):735. doi: 10.3390/bs13090735 (PMC10525546; doi:10.3390/bs13090735)
Supplement: Supplementary file 1 [file behavsci-13-00735-s001.zip › behavsci-2567661-supplementary.pdf]

Report on Covid-19

Week 24–30 *December*

During the two weeks of the Christmas holidays, in which there was a significant increase in the number of Covid-19 positive cases, the cohesion degree increased by almost one percentage point from the previous survey, from a value of 10.98 to 11.24. What allows the Hyperion Observatory to observe this fluctuation is the increase of almost three percentage points in the Common References and Shared Responsibility clusters (from 29.57% to 32.89%) and the decrease of an equal number of points in the Conflict and Dispute clusters, the sum of which goes from 70.43% to 67.11%.
In the light of the above, two scenarios emerge in detail in the textual data analyzed:

A) the vaccine is seen as a tool for citizens to anticipate the increase in contagion and its implications (i.e. increased sanitary restrictions, use of PPEs, quarantine timelines) and thus be prepared to manage and use them in pursuit of the common goal of reducing the spread of contagion. In fact, if the value with which the vaccine is configured tends to be that of a tool for reducing the spread of contagion, then one is in a position to contemplate as possible and, therefore, manage an eventual increase in the number of positive cases and an eventual increase in sanitary restrictions.
B) On the other hand, the textual data analyzed by Hyperion show that considering the vaccine as a 'solution to the problem' of the sanitary emergency opens up the possibility of configuring the increase in infections as a 'failure' of the drug itself. This is highlighted by 33.63% of the textual data analyzed, which shows the tendency of the Veneto region's citizens to comment on the ineffectiveness of the vaccine (given the increase in the number of Covid-19 positive cases recorded in the Civil Protection reports) and to judge negatively the institutions that promoted the campaign. The use of this modality exacerbates the conflict (already present for months) between a part of the citizens and the institutions (as can be seen from the value of Dispute – 33.63% – which, although decreasing, remains high).

Week 31 December – 6 January

https://infogram.com/bollettino-coesione-sociale-31-dicembre-6-gennaio-2022-1h7z2l8kpnz9x6o?live
